# The mediating roles of buoyancy and boredom in the relationship between autonomous motivation and engagement among Chinese senior high school EFL learners

**DOI:** 10.3389/fpsyg.2022.992279

**Published:** 2022-10-17

**Authors:** Ying Wang, Honggang Liu

**Affiliations:** ^1^School of Foreign Languages, Northeast Normal University, Changchun, China; ^2^School of Foreign Languages, Soochow University, Suzhou, China

**Keywords:** autonomous motivation, engagement, boredom, buoyancy, EFL learners

## Abstract

For several decades, there has been an increase in studies on second language motivation, one of the most salient topics in individual difference research in second language acquisition, guided by theories and methods from related fields. Self-determination theory (SDT) is one of the most influential theories to provide a comprehensive framework for investigating language learning motivation. To date, numerous SDT-related studies have been performed to explore ways to develop more self-determined types of motivation. However, research on the relationship between self-determined types of motivation and other psychological variables has been limited. To address this gap, the present study investigated the complex relationships between autonomous motivation, buoyancy, boredom, and engagement in a sample of 561 Chinese senior high school English as a foreign language (EFL) learners. Data were collected through a composite questionnaire measuring students’ autonomous motivation, buoyancy, boredom, and engagement in EFL learning. Chain mediation analysis was used to test the complex relationships among these variables. The results show that autonomous motivation directly affected student engagement in EFL learning and autonomous motivation also indirectly affected student engagement in EFL learning through the separate mediation of buoyancy and boredom in EFL learning as well as the chain mediation of both mediators. The results support SDT and offer some pedagogical implications for teachers and educators.

## Introduction

Individual difference (ID) research is a firmly established field of inquiry in second language acquisition (SLA) studies (Ellis, 2008; Dörnyei, 2009). Recently, there has been a plethora of studies on various ID factors among English as a foreign language (EFL) teachers and learners (e.g., Shao et al., 2020; Li and Liu, 2021; Liu and Song, 2021; Chu and Liu, 2022; Gao et al., 2022; Liu and Chu, 2022; Shirvan and Alamer, 2022). EFL motivation, a key individual difference variable influencing effective language learning (Dörnyei and Ryan, 2015), has interested researchers for several decades. The introduction of positive psychology (MacIntyre and Mercer, 2014) led to an increase in the number of studies focusing on foreign language learning motivation as one of the most important themes in research on language learners’ well-being (Oxford, 2016). Theories and approaches in related fields, such as psychology and sociology, have made interesting progress. Self-determination theory (SDT; Deci and Ryan, 1985) is among the most influential theories used to understand human motivation and functioning that has been applied to many research domains, including SLA, as it provides a comprehensive framework for investigating language learning motivation (Sugita-McEown and Oga-Baldwin, 2019; Noels et al., 2020). Specifically, since the 1990s, SLA researchers have been adopting SDT to explore language learners’ situated language learning environments and basic psychological needs in connection with more self-determined types of motivation (i.e., intrinsic motivation and identified regulation) and better learning achievement (e.g., Noels et al., 1999; Pae and Shin, 2011; Henry, 2017; Shelton-Strong, 2020; Alrabai, 2021).

In recent years, SLA research has experienced an affective turn (Pavlenko, 2013). Accordingly, in addition to focusing on how to initiate and sustain more self-determined types of motivation in learners, a host of SDT-related studies has examined the relationships between EFL motivation and other psychological variables and shown its significant association with student engagement (e.g., Chen and Kraklow, 2015; Oga-Baldwin and Nakata, 2017; Tsao et al., 2021), anxiety (e.g., Khodadady, 2013; Alamer and Almulhim, 2021), self-confidence (e.g., Lou and Noels, 2021), and willingness to communicate (e.g., Peng and Woodrow, 2010; Joe et al., 2017; Lin, 2019). However, there is a paucity of research on the relationships between self-determined motivation and positive character strengths (e.g., academic buoyancy) as well as emotions other than anxiety (e.g., boredom), which have emerged as salient topics in current SLA research. Therefore, this study aimed to comprehensively investigate the relationships between self-determined motivation, buoyancy, boredom, and engagement using the framework of SDT.

## Literature review

### Self-determination theory

SDT (Deci and Ryan, 1985; Ryan and Deci, 2017) is a macro theory of human motivation that highlights the extent to which an individual’s basic psychological needs are satisfied as a function of interpersonal and social dynamics and it determines types of human motivation and thus flourishing. Since SDT was proposed in the 1980s, it has been expanded into a macro theory of human motivation comprising six mini-theories, namely, cognitive evaluation theory, organismic integration theory, causality orientations theory, basic psychological needs theory, goal contents theory, and relationships motivation theory.

Organismic integration theory (OIT) is a mini-theory of the greatest interest (Al-Hoorie et al., 2022). It classifies motivation into three types, namely, amotivation, extrinsic motivation, and intrinsic motivation. Amotivation refers to the state of lacking intention or motivation. Extrinsic motivation describes human behaviors that are initiated because of an external reward or social approval, avoidance of punishment or attainment of a valued outcome. It is further differentiated into varied forms in terms of the degree of self-determination. They are external regulation, introjected regulation, identified regulation, and integrated regulation, ranked from least to most autonomous. Integrated regulation involves the internalization of external rules and regulations as part of one’s identity. However, integrated regulation is often not attained, especially for children and teenagers or people with limited experience in undertaking a certain task (Vallerand et al., 1989, as cited in Noels et al., 1999). Consequently, it is overlooked in most studies on SDT motivation in education. A person with identified regulation is characterized as perceiving the outcomes a behavior yields as personally important. By contrast, if people’s behavior is triggered by introjected regulation, it is associated with personal responsibility or pressure. In external regulation, behaviors are carried out because of external rewards or punishments. Another type of motivation is intrinsic motivation, which is in contrast with extrinsic motivation and refers to the behaviors initiated because of personal interest, fun, and satisfaction.

The above types of motivation constitute a self-determined continuum from non-regulation, to controlled regulation, to autonomous regulation (Ryan and Deci, 2000). Consequently, motivation can be studied by examining more general types of autonomous and controlled motivation (Ryan and Deci, 2017, 2020). Intrinsically motivated behaviors are autonomous because they are performed out of enjoyment and interest. Behaviors can also be autonomous through extrinsic motivation. In extrinsic motivation, regulations through integration and identification are more autonomous, whereas external and introjected regulation represent controlled types of motivation. In addition to maintaining the existence of the above subtypes of motivation, OIT rationalizes the antecedents and outcomes of these motivation types. Autonomous types of motivation are facilitated by support for an individual’s basic psychological needs for autonomy, competence, and relatedness. In turn, they are associated with several positive outcomes regarding people’s achievement, psychological growth, and wellness. The controlled types of motivation, however, arise from basic psychological needs and frustration and are related to less positive outcomes (Ryan and Deci, 2017). In summary, SDT emphasizes human growth and wellness and has strong implications for various domains.

In language learning, it is more important to build autonomous types of motivation to stimulate students to voluntarily participate in language learning activities to improve the quality of learning (Sugita-McEown and Oga-Baldwin, 2019). However, in addition to exploring the ways of promoting students’ autonomous motivation, it is of great significance to understand the influencing mechanism underlying autonomous motivation and outcome variables, which has received limited attention in previous studies. Therefore, we focused on the autonomous form of motivation and its impact on learning engagement in EFL learning. Thus, the dichotomous classification of autonomous and controlled motivation provides a clear theoretical framework for understanding students’ autonomous motivation as it involves both intrinsic motivation and identified type of extrinsic motivation in one framework.

### Antecedents and outcomes of autonomous motivation in EFL learning

With respect to SDT research on SLA, Noels et al.’s (1999) research is noteworthy as they were the first to apply SDT to explore second language (L2) motivation and classify it into intrinsic motivation, extrinsic motivation, and amotivation, which was well confirmed in subsequent studies (e.g., Noels et al., 2000; Noels, 2001). In view of notable works by Noels and her colleagues, as well as research in educational psychology (e.g., Ryan and Connell, 1989), another sequence of studies further explored the EFL motivation construct. Oga-Baldwin and Nakata (2017) verified the four-dimensional motivation construct in the EFL learning context, which was first developed by Ryan and Connell (1989), among Japanese primary school EFL learners. This construct was further substantiated by Alamer (2021a) among Arabic university students. Alamer (2021a) also established the existence of two overarching constructs – that is, autonomous motivation and controlled motivation – which Alamer (2021b) later confirmed using the advanced bifactor-exploratory structural equation modeling method.

The growing body of research in various cultural and language learning contexts has consistently suggested that autonomy-supportive learning environments are conducive to autonomous motivation to learn EFL, which further affects learning behaviors and achievements. For example, Noels et al. (1999) investigated the relationship between French as a second language learners’ perceptions of teacher communicative style, SDT motivation, and emotional, motivational, and competence variables in the Canadian context and found that learners who felt their teacher was informative and their learning environment was less controlling manifested more autonomous motivation, which was related to a lower level of anxiety and higher degree of motivational intensity, the intention to continue L2 study and self-evaluation of competence. Pae and Shin’s (2011) study demonstrated this as well. Their survey revealed that the autonomy-supportive communicative teaching method among South Korean university EFL students profoundly affected intrinsic motivation and its relation to a set of psychological factors and achievement, such as self-confidence and achievement. Another example was offered by Joe et al. (2017), whose questionnaire explicated the significant indirect role of English classroom social climate among South Korean secondary school students on their autonomous motivation and also suggested that identified regulation was predictive of willingness to communicate.

The above studies were primarily interested in the role of autonomy-supportive learning environments and learner internal factors in the development of students’ autonomous motivation. They also considered the relationship between autonomous motivation and other psychological and learning outcome variables, such as motivational intensity and willingness to communicate. The researchers additionally explored the relationship between autonomous motivation and engagement in EFL learning.

Comanaru and Noels (2009) investigated the relationship between SDT motivation, basic psychological needs, engagement in learning, and community engagement among Chinese as heritage language learners and found that learners with intrinsic motivation and identified regulation were inclined to engage more in the learning process. Chen and Kraklow (2015) further demonstrated the strong predictive power of intrinsic motivation on English learning engagement among Taiwanese college students. They investigated the differences in SDT motivation and engagement among students attending English as the medium of instruction (EMI) and non-EMI programs as well as the predictive role of SDT motivation in engagement. Their results showed the major predictive role of intrinsic motivation in students’ engagement. The relationship was also tested in English writing instruction. Tsao et al. (2021) exhibited that Chinese undergraduate students’ intrinsic motivation to learn English writing was predictive of students’ engagement in written corrective feedback.

In recent years, guided by SDT and the four-dimensional engagement framework developed by Reeve and Tseng (2011), involving behavioral, affective, cognitive, and agentive engagement, studies have begun to evaluate the relationship between motivation and engagement in EFL learning. One of these studies was that of Dincer et al. (2019), who investigated the antecedents and outcomes of engagement among Turkish university EFL learners. The results indicated a linear causal relationship between teachers’ autonomy support, students’ needs satisfaction, engagement, and achievement/absenteeism within English courses. These results revealed the possible significant predictive role of autonomous motivation in engagement by testing the proximal influence of basic psychological needs satisfaction on engagement.

### Mediating role of buoyancy

Academic buoyancy is defined as ‘students’ ability to successfully deal with academic setbacks and challenges that are typical of the ordinary course of school life’ (Martin and Marsh, 2008, p. 54) and has gradually become a point of interest in the general education and SLA fields. In general education research, Martin and Marsh (2008) proposed the one-dimensional academic buoyancy framework, which offers researchers a scientific research framework and assessment tool for examining students’ subject-specific buoyancy (e.g., Malmberg et al., 2013; Collie et al., 2015, 2017; Datu and Yang, 2019; Aydın and Michou, 2020). Turning to the structure of buoyancy in the SLA domain, most studies, with only a few exceptions (e.g., Jahedizadeh et al., 2019, 2021; Yang et al., 2022), have delved into buoyancy in EFL learning based on the one-dimensional framework used in the general education field. For instance, Yun et al. (2018) adapted one-dimensional instrument from Martin and Marsh’s (2008) to measure buoyancy among South Korean university EFL learners and revealed that buoyancy significantly predicted both English and general academic achievement and mediated the effects of self-efficacy, self-regulation, ideal L2 self, and teacher–student relationship on two achievement variables.

In terms of the relationship between autonomous motivation and buoyancy in EFL learning, Aydın and Michou (2020) suggested that university EFL learners’ autonomous motivation was predictive of their buoyancy, which further influenced their EFL achievement. Owing to the significant predictive role of engagement in learning performance (Finn and Zimmer, 2012), buoyancy would likely predict student engagement in EFL learning. The possible link between buoyancy and engagement in EFL learning was supported by a handful of studies performed in the general education field. An example includes the study of Martin (2014), who investigated academic buoyancy among high school students with ADHD and demonstrated that academic buoyancy predicted students’ cognitive, affective and behavioral engagement. Another example is the study by Af Ursin et al. (2021), which also confirmed the predictive role of academic buoyancy in primary school students’ affective and cognitive engagement. However, students may encounter many setbacks and challenges, including poor exam results, learning plateaus, and negative feedback, in senior high school EFL learning (Liu and Han, 2022). Thus, it is worth investigating whether students’ autonomous motivation predicts their buoyancy in EFL learning, which in turn affects their engagement in such a stressful environment. In other words, it is possible that buoyancy in EFL learning mediates the relationship between autonomous motivation and engagement among senior high school EFL learners.

### Mediating role of boredom

Emotions profoundly affect EFL learning and performance (Swain, 2013). Since the 1980s, there has been a dramatic increase in attention to anxiety in EFL learning, involving varied antecedents and outcomes of teacher and learner anxiety in different educational contexts (e.g., Horwitz et al., 1986; Shao et al., 2013; Liu et al., 2022). Empirical findings corroborated that autonomous motivation negatively predicted anxiety in EFL learning (Noels et al., 1999; Khodadady, 2013; Alamer and Almulhim, 2021), which in turn negatively affected student engagement (Zhang et al., 2020; Wang et al., 2021). However, apart from a few exceptions (e.g., Ross, 2015), studies on negative emotions other than anxiety in EFL learning were slow to emerge. Therefore, researchers suggested adopting interdisciplinary theories and methods, such as the control-value theory of achievement emotions (Shao et al., 2019, 2020; Shao and Parkinson, 2021) and sentiment analysis (Lei and Liu, 2021), to explore a wider range of emotions in EFL learning and use.

Boredom represents ‘the aversive experience of having an unfulfilled desire to be engaged in satisfying activity’ (Fahlman et al., 2013, p. 69). It is a key emotion that EFL researchers have begun to recognize in recent years (e.g., Pawlak et al., 2020c; Derakhshan et al., 2021). Concerning its conceptualization, Pekrun et al. (2005, 2011) studied class-related boredom and learning-related boredom in the field of education. The conceptualization was applied in many EFL boredom studies investigating the links between boredom and other variables. For example, Dewaele and Li (2021) reformulated Pekrun et al.’s (2011) framework to fit the context of university EFL learning and found that students’ boredom positively predicted their social-behavioral engagement; furthermore, students’ boredom mediated the relationship between perceived teacher enthusiasm and social-behavioral engagement. Moreover, a few studies (e.g., Derakhshan et al., 2022) have employed EFL subject-specific research frameworks (e.g., Pawlak et al., 2020c; Shirvan et al., 2021) to explore the relationship between EFL learners’ boredom and other factors and observed the negative influence of boredom on engagement. Additionally, several studies have identified other antecedents of boredom through qualitative approaches, such as learning attitudes (e.g., Pawlak et al., 2020a, 2020b). However, little is known about the predictive role of autonomous motivation in boredom or the effect of boredom on engagement.

### The relationship between buoyancy and boredom

In terms of the relationship between buoyancy and boredom, researchers have investigated the link between these two factors as well as the mediating role of boredom between buoyancy and learning-related expectations and behaviors. For instance, Hirvonen et al. (2019) explored the role of emotions and academic buoyancy in the formation of failure expectation, avoidance behavior, and task-oriented planning among Finnish primary school students. The results showed that academic buoyancy predicted boredom and boredom mediated the relationship between academic buoyancy and failure expectation together with avoidance behavior. Nevertheless, little is known about how academic buoyancy relates to boredom in the EFL learning context.

### The present study and research hypotheses

**Figure 1:**
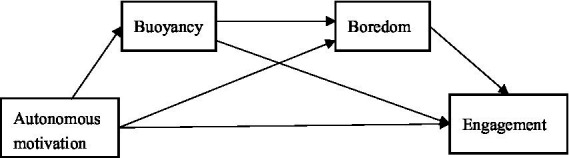
Hypothesized Model.

SDT and relevant research in the general education and EFL research fields provide strong support for the present study. On one hand, SDT maintains that students’ autonomous motivation plays a positive predictive role in positive learning psychology and behaviors (Deci and Ryan, 1985; Ryan and Deci, 2017). On the other hand, SDT-related findings empirically support the possible direct or indirect complex effects that the autonomous motivation of EFL students has on various aspects of their engagement. Specifically, the research thus far has identified a direct link between autonomous motivation and engagement in EFL learning (e.g., Comanaru and Noels, 2009; Dincer et al., 2019) and an indirect link between them through the intermediate variable of anxiety in EFL learning (e.g., Alamer and Almulhim, 2021; Wang et al., 2021). However, the mediating role of boredom, which is a ubiquitous negative emotion experienced by EFL learners (Pawlak et al., 2020c), has not been explored. In addition, buoyancy research in the general education field has indicated a possible mediating role of buoyancy in the relationship between autonomous motivation and engagement in EFL learning (e.g., Martin, 2014; Af Ursin et al., 2021) as well as the relationship between buoyancy and boredom (e.g., Hirvonen et al., 2019), which is scant in the EFL context. Thus, further examination of the mediating role of buoyancy in the relationship between autonomous motivation and engagement and the predictive role of buoyancy in boredom in the context of EFL learning is needed, especially in the Chinese senior high school EFL learning context, where students’ buoyancy profoundly impacts their learning (Liu and Han, 2022). Taken together, the present study aimed to fill the gap in the literature by exploring the complex influencing mechanisms underpinning the link between autonomous motivation and student engagement among Chinese senior high school EFL learners (see Figure 1). The hypothesized model and concrete hypotheses are as follows:

*Hypothesis 1*: Autonomous motivation directly affects student engagement in EFL learning.

*Hypothesis 2*: Autonomous motivation indirectly affects student engagement in EFL learning, mediated through buoyancy.

*Hypothesis 3*: Autonomous motivation indirectly affects student engagement in EFL learning, mediated through boredom.

*Hypothesis 4*: Autonomous motivation indirectly affects student engagement in EFL learning, mediated through buoyancy and boredom.

## Research design

### Participants

A sample of 561 Chinese senior high school students participated in the study. They were from five senior high schools located in five cities in three provinces and one autonomous region. The numbers of male and female participants were 239 (42.6%) and 322 (57.4%), respectively. The sample comprised 308 Grade 1 students, 194 Grade 2 students, and 59 Grade 3 students. The sample presented diversity in school achievement. All participants were learning EFL.

### Instruments

We used a questionnaire to collect data on the students’ background information (i.e., sex, grade, school name, and English achievement score) and their autonomous motivation, buoyancy, boredom, and engagement in EFL learning. All items in the questionnaire used a 5-point Likert scale ranging from 1 (strongly disagree) to 5 (strongly agree). The items were translated into Chinese by the author. Two MTI (English) students were invited to improve the items’ language quality.

#### Autonomous motivation

We used the Autonomous Motivation Subscale in the Self-Determination Theory in Second Language Scale (Alamer, 2021a) to measure the participants’ autonomous motivation to learn EFL. The scale includes 10 items, such as ‘Because I enjoy learning English’ and ‘Because people around me (teacher/peers/parents) expect me to learn English’.

#### Buoyancy

We also adapted the Academic Buoyancy Scale compiled by Yun et al. (2018) to measure participants buoyancy in EFL learning. The scale includes four items, such as ‘Once I decide to do something for English learning, I am like a bulldog: I do not give up until I reach the goal’ and ‘In English class, I continue a difficult task even when the others have already given up on it’.

#### Boredom

We adapted the Boredom Subscale in the Academic Emotion Questionnaire-Short Form developed by Bieleke et al. (2021) to measure boredom in EFL learning. The scale includes eight items, and they were revised to fit the current study. For example, the item ‘I get bored’ in the original scale was restated as ‘I get bored in English class’.

#### Engagement

To measure the participants’ four aspects of engagement in EFL learning, we also adapted the Student Engagement Scale developed by Reeve and Tseng (2011). The scale includes 22 items, and they were revised to fit the current study. For example, the item ‘I listen carefully in class’ in the original scale was revised to ‘I listen carefully in English class’.

### Procedure

After the teachers and participants agreed to support the study, participants received a composite questionnaire in December 2021. All scales were uploaded to the online survey tool,^1^ and its web address was provided to the participants during the online classes. Altogether, there were 561 responses. There were 519 valid responses after the data were screened in terms of invariant responses to the questionnaire items. First, we used SPSS 24.0 and Mplus 7 statistical software to process the data. Preliminary data analysis employed confirmatory factor analysis to test the psychometric properties of the measurement model. Descriptive and Pearson correlation analyses were conducted to analyze the general characteristics of the research variables. In the main analysis, we used PROCESS v4.0 (Model 6) developed by Hayes (2018) to test the hypothesized model, calculating the path coefficients and direct and indirect effects between variables and presenting corresponding bootstrap confidence intervals.

## Results

### Preliminary analyses

The measurement model with four factors—namely, autonomous motivation, buoyancy, boredom, and engagement—was tested. The first round of confirmatory factor analysis showed that the four-factor measurement model did not fit the data well (χ^2^/df = 4.67, RMSEA = 0.084, SRMR = 0.065, CFI = 0.848, TLI = 0.839). After three rounds of modification, one item from autonomous motivation and two items from engagement factor were discarded because of low factor loadings, and the four-factor measurement model with 41 items yielded an adequate fit (χ^2^/df = 3.41, RMSEA = 0.068, SRMR = 0.054, CFI = 0.907, TLI = 0.900). The standardized estimates of factor loadings for the constructs ranged from 0.57 to 0.93. Cronbach’s alphas for the four factors were 0.87, 0.89, 0.89 and 0.85, respectively. The above results suggested good construct validity and reliability of the four-factor measurement model.

Table 1 presents the means and standard deviations of autonomous motivation, boredom, buoyancy, and engagement together with the results of the Pearson correlation analysis among these variables. The descriptive analysis showed that the participants were autonomously motivated to learn EFL (*M* = 3.75, *SD* = 0.77). In addition, they were inclined to be buoyant (*M* = 3.58, *SD* = 0.90) and highly engaged (*M* = 3.64, *SD* = 0.72), and they felt less bored in learning EFL (*M* = 2.00, *SD* = 0.87).

**Table 1:** Results of descriptive statistics and interrelations among variables.

	*M*	*SD*	Moa	Bor	Buo	Eng
Moa	3.75	0.77	–			
Bor	2.00	0.87	−0.576^**^	–		
Buo	3.58	0.90	0.600^**^	−0.533^**^	–	
Eng	3.64	0.72	0.652^**^	−0.543^**^	0.822^**^	–

*N* = 519, **p < 0.01.

Moa, autonomous motivation; Bor, boredom; Buo, buoyancy; Eng = engagement

Regarding correlations between the variables under investigation, we found a significant correlation between autonomous motivation, buoyancy, boredom, and engagement (*p* < 0.01). In this regard, autonomous motivation was negatively and significantly correlated with boredom (*r* = −0.576, *p* < 0.01) but positively and significantly correlated with buoyancy and engagement (*r* = 0.600, *p* < 0.01; *r* = 0.652, *p* < 0.01). Boredom was negatively and significantly correlated with buoyancy and engagement (*r* = −0.533, *p* < 0.01; *r* = −0.543, *p* < 0.01). Buoyancy was positively and significantly correlated with engagement (*r* = 0.822, *p* < 0.01).

### Test of the mediating roles of buoyancy and boredom between autonomous motivation and engagement

**Figure 2:**
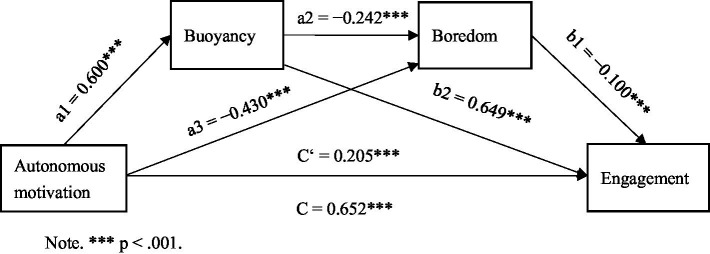
Path diagram of the mediation model. ***p < 0.001.

Based on the aforementioned hypothesized model, we tested the mediating effects of buoyancy and boredom on the relationship between autonomous motivation and engagement among senior high school EFL learners using PROCESS v4.0 (Model 6) with 5,000 random-sample bootstrapping confidence intervals (CIs). The tested mediating model is presented in Figure 2. All path coefficients between the variables under investigation were significant (*p* < 0.001). In addition, the direct and indirect mediating effect sizes as well as the corresponding bootstrap CIs are displayed in Table 2.

**Table 2:** Direct and indirect effects of autonomous motivation on engagement through boredom and buoyancy.

Pathway	Estimate	SE	95% CIs	Relative effect (%)
Direct effect	0.193	0.030	[0.135, 0.252]	31.38
Total indirect effect	0.422	0.422	[0.344, 0.506]	68.62
Moa → Buo → Eng	0.368	0.041	[0.288, 0.446]	59.84
Moa → Bor → Eng	0.041	0.019	[0.007, 0.080]	6.67
Moa → Buo → Bor → Eng	0.014	0.007	[0.002, 0.030]	2.28

Moa = autonomous motivation; Bor = boredom; Buo = buoyancy; Eng = engagement

As presented in Table 2, both the direct effect of autonomous motivation on engagement and the total indirect effect of autonomous motivation on engagement through the two mediators, buoyancy and boredom, were significant, because the 95% CIs did not include zero (from 0.135 to 0.252; from 0.344 to 0.506, respectively). The direct effect size was 0.193, accounting for 31.38% of the total effect of autonomous motivation on engagement, and the total indirect effect size was 0.422, accounting for 68.62% of the total effect of autonomous motivation on engagement. Therefore, buoyancy and boredom served partial mediating functions in the relationship between autonomous motivation and engagement in EFL learning among senior high school learners.

Concerning the indirect effect, the indirect effect sizes of autonomous motivation on engagement through the two mediators, buoyancy and boredom, were 0.368 and 0.041, respectively. The corresponding 95% CIs (from 0.288 to 0.446; from 0.007 to 0.080, respectively) indicated that both mediating paths were significant. Regarding the indirect effect of autonomous motivation on engagement through both buoyancy and boredom, the 95% CI (from 0.002 to 0.030) indicated that it was also significant. The effect size was 0.014, accounting for 2.28% of the total effect of autonomous motivation on engagement. Taken together, all four hypotheses proposed above were empirically validated.

## Discussion

The current study explored the complex relationships between autonomous motivation, buoyancy, boredom, and engagement among Chinese senior high school EFL learners and identified the significant direct and indirect effects of autonomous motivation on engagement through the mediators of buoyancy and boredom in EFL learning.

### The direct effect of autonomous motivation on engagement

First, the results show that students’ autonomous motivation could positively predict their engagement in EFL learning, verifying Hypothesis 1. According to SDT, intrinsic motivation represents the most self-determined type of motivation that improves the quality of students’ engagement in learning (Ryan and Deci, 2017). Similarly, the identified regulation is also characterized by a relatively high degree of active engagement in that learning activities are consciously valued and thus are more congruent with the students’ core values, goals, and interests. Additionally, the current result is supported by previous studies. Comanaru and Noels (2009) found that autonomously motivated heritage language learners demonstrated more effort in learning the language. Likewise, intrinsically motivated university EFL learners were able to properly concentrate on learning activities (Chen and Kraklow, 2015). The result is also supported by those of Dincer et al. (2019), who found that university EFL learners’ behavioral, cognitive, affective, and agentive engagement was directly predicted by satisfaction of their basic psychological needs. In view of the close relationship between basic psychological needs satisfaction and autonomous motivation (Ryan and Deci, 2017, 2020), we can conclude that autonomous motivation is directly and positively related to the quality of student engagement in EFL learning. More precisely, autonomously motivated senior high school EFL learners are those whose motivation is based on the inherent satisfaction and enjoyment they feel in EFL learning or on the conscious identification of the value that EFL learning expresses, such as offering new opportunities for better career development. Therefore, autonomously motivated students engage in EFL learning with more energy, enthusiasm, and active responses to the learning environment to achieve better development.

### The mediating role of buoyancy in the relationship between autonomous motivation and engagement

Results of the present study also suggest that the mediating role of buoyancy in the relationship between autonomous motivation and engagement in EFL learning was significant, confirming Hypothesis 2. According to SDT, in acting out of intrinsic motivation, students are driven by self-growth and are likely to focus on challenges to express their interest and expand their skills and knowledge (Ryan and Deci, 2017). Similarly, more internalized extrinsic motivation (i.e., identified motivation) is associated with greater persistence and proactive coping (Pelletier et al., 2001; Ryan and Deci, 2017), which is essential to higher learning performance. Moreover, the mediating role of buoyancy in EFL learning is supported by previous findings. As Aydın and Michou (2020) explained, university EFL learners with higher autonomous motivation tend to show greater academic buoyancy. Chaffee et al. (2014) also found a significant positive correlation between autonomous motivation and indicators of resilience. Additionally, research on buoyancy in EFL learning following other frameworks (e.g., L2 Motivational Self-System) supported the current finding. According to Yun et al. (2018), university students’ ideal L2 self was predictive of buoyancy in English learning. Considering the view that students with a higher level of ideal L2 self tend to be fully aware of the benefits of L2 learning, which enforces the identified regulation to learn L2 (Takahashi and Seongah, 2020), it is probable that EFL learners who are autonomously motivated tend to be more buoyant in learning. Correspondingly, students with a higher level of buoyancy in EFL learning tend to have a higher level of engagement (Martin, 2014; Thomas and Allen, 2021). Although nearly all the aforementioned studies on buoyancy in EFL learning were conducted in the university context, they still contributed to the results of the present study. That is, the autonomous motivation of senior high school EFL learners indirectly affected their engagement in EFL learning through buoyancy.

### The mediating role of boredom in the relationship between autonomous motivation and engagement

Consistent with Hypothesis 3, results of this study indicate that EFL boredom mediated the relationship between autonomous motivation and engagement in EFL learning. According to SDT-related research (e.g., Ryan and Connell, 1989), autonomous types of motivation displayed a strong correlation with positive emotions (e.g., enjoyment) but showed no correlation or a very weak correlation with negative emotions (e.g., anxiety). Kong and Liu (2020) and Liu et al. (2021) obtained similar findings that autonomously motivated secondary school learners were likely to experience a higher level of enjoyment and lower level of boredom. The predictive role of autonomous motivation in boredom in EFL learning is also supported by previous studies in the SLA field. For example, the studies of Noels et al. (1999), Khodadady (2013) and Alamer and Almulhim (2021) converged on the fact that the higher the autonomous motivation, the lower the learning anxiety among EFL learners in both the high school and university contexts. In turn, a lower level of anxiety in EFL learning would predict more active student engagement, such as deep processing and active participation in classroom activities (Zhang et al., 2020; Wang et al., 2021). By the same token, it is sound to claim that boredom could mediate the relationship between autonomous motivation and engagement among senior high school EFL learners as a result of the same impeding role of boredom and anxiety in L2 learning (MacIntyre and Gardner, 1994; Khajavy et al., 2018; Pawlak et al., 2020c). To put it another way, when senior high school students were autonomously motivated to learn EFL, they were less likely to experience boredom, more likely to be engaged in EFL learning by interacting with their teacher and peers (Dewaele and Li, 2021), and more likely to apply various motivational strategies to improve the quality of learning.

### The chain mediating role of buoyancy and boredom in the relationship between autonomous motivation and engagement

The most important finding of the present study is that the indirect effect of autonomous motivation on engagement in EFL learning through the chain mediating role of buoyancy and boredom was also significant, supporting Hypothesis 4. The results indicate that EFL learners in senior high school who were learning out of interest, enjoyment, or a sense of value expressed a higher ability to bounce back from everyday learning setbacks and correspondingly experienced a low level of boredom, thus contributing to active engagement in EFL learning. This result is in line with the general education research literature. As Hirvonen et al. (2019) and Dewaele and Li (2021) indicated, students’ high buoyancy was related to low boredom, which in turn affected students’ engagement in learning. Although limited research attention has been given to the mediating role of boredom in the relationship between academic buoyancy and engagement, several studies have focused on the relationship between academic buoyancy and negative emotions as well as the relationship between negative emotions and engagement. For example, Putwain et al.’s (2015) longitudinal study indicated that the cognitive component of anxiety, worry, predicted academic buoyancy, and academic buoyancy also predicted worry. Additionally, Martin et al.’s (2010) investigation demonstrated the predictive role of the reciprocal relationship between academic buoyancy and general anxiety, which further predicted students’ engagement in learning (Zhang et al., 2020; Wang et al., 2021). Given the positive relationship between anxiety and boredom, on one hand, it is reasonable to claim the negative predictive role of buoyancy on boredom; on the other hand, it is appropriate to declare the mediating role of boredom in the relationship between academic buoyancy and engagement in EFL learning. In addition, SDT (Ryan and Deci, 2017) and the relevant research in SLA (e.g., Aydın and Michou, 2020) consistently indicated the significant predictive role of autonomous motivation in academic buoyancy. In summary, we conclude that if senior high school EFL learners are autonomously motivated, they are inclined to actively seek ways to overcome adversities and difficulties in EFL learning, and they in turn perceive less boredom, which further improves their level of engagement.

## Implications and limitations

The results of the current study confirm the significant mediating roles of buoyancy and boredom in the link between autonomous motivation and student engagement among Chinese senior high school EFL learners and suggest that autonomous motivation could be an important antecedent of buoyancy, boredom, and engagement. Therefore, to improve the engagement of Chinese senior high school EFL learners, teachers can show concern for enhancing students’ autonomous motivation to learn English.

More specifically, first, students’ autonomous motivation should be cultivated through the optimization of teaching resources and teaching methods. Stimulating an interest in learning and promoting a sense of satisfaction could be conducive to reducing boredom and promoting engagement. The current senior high school EFL class is characterized by an overemphasis on knowledge input in class (e.g., large vocabulary and grammar exercises). Consequently, students may experience monotony and dissatisfaction, which are detrimental to fostering autonomous motivation for EFL learning. This situation could be averted by improving teaching content by combining the use of textbooks with other resources, such as multimodal resources, students’ everyday lives, and current events, and by applying information technology to alter traditional teaching modes and enrich class activities. These teaching practices may also help in meeting every student’s learning needs, which would be constructive in strengthening their buoyancy in dealing with everyday learning setbacks and enhance their engagement. Second, few students can identify the value of learning EFL, which also hampers the development of autonomous motivation. Studying EFL is needed not only to obtain better grades on the college entrance examination but also for self-development, which is an essential skill for future learning and work. However, quite a few senior high school students were forced to learn EFL because of exams and demands from others. These students are prone to be less buoyant and experience more boredom, leading to passive engagement. Therefore, it is equally important to foster the value of learning EFL among students.

The present study faced several limitations. First, we used a cross-sectional design to investigate the complex relationships between autonomous motivation, academic buoyancy, boredom, and engagement among Chinese senior high school EFL learners. The generalizability of the results should be further examined in future studies by utilizing a longitudinal research design or replicating this study in other cultural or learning contexts. In particular, concerning the relationship between buoyancy and boredom, studies (e.g., Azadianbojnordi et al., 2020) have suggested the predictive role of other emotions (e.g., hope) in buoyancy. Therefore, it is significant to further explore the chain mediating role of boredom in buoyancy in the relationship between autonomous motivation and engagement in EFL learning. Second, controlled motivation is an important type of motivation theorized in SDT. However, this study did not research this due to time and energy constraints. Therefore, both autonomous and controlled motivation should be taken into account in future studies. Third, this study, based on SDT, validated the chain mediating role of EFL buoyancy and boredom in the relationship between autonomous motivation and engagement in EFL learning. However, a few studies (e.g., Putwain et al., 2020, 2022) have indicated a moderating effect of academic buoyancy on the relationship between emotions and adaptive and maladaptive learning behaviors. Therefore, it is also worth exploring the moderating effect of buoyancy in EFL learning on SDT motivation, boredom, and engagement.

## Conclusion

Based on combined empirical evidence and SDT formulations, the present study tested a hypothesized structural model concerning senior high school students’ autonomous motivation, buoyancy, boredom, and engagement in EFL learning. The findings indicate that students’ autonomous motivation to learn EFL affects their engagement directly and indirectly through the separate mediation of buoyancy and boredom in EFL learning as well as the chain mediation of these two mediators. The exploration of the direct and indirect paths in the link between autonomous motivation and student engagement in EFL learning is notable as it extends the knowledge of SDT in the SLA domain. To the best of our knowledge, the present study is the first to explore the mediating roles of buoyancy and boredom in the relationship between autonomous motivation and student engagement in the EFL learning context. It indicates that autonomously motivated senior high school EFL learners are more capable of bouncing back from everyday learning setbacks and feel less bored, which promotes students’ engagement in learning. Therefore, it is considered crucial for senior high school EFL educators to enhance students’ autonomous motivation to learn in the EFL classroom.

## Data availability statement

The original contributions presented in the study are included in the article/supplementary material, further inquiries can be directed to the corresponding author.

## Author contributions

YW conceptualization, data analysis, writing, and revision. HL conceptualization, data analysis, revision, supervision, and funding. All authors contributed to the article and approved the submitted version.

## Funding

This paper was supported by the Project of Discipline Innovation and Advancement (PODIA) – Foreign Language Education Studies at Beijing Foreign Studies University (Grant number: 2020SYLZDXM011) and the Project of Developmental Features of the Language Ability by Chinese Multilingual Second Language Learners-Youth Team Funding of Northeast Normal University, 2021 (Grant number: 2021QT004).

## Conflict of interest

The authors declare that the research was conducted in the absence of any commercial or financial relationships that could be construed as a potential conflict of interest.

## Publisher’s note

All claims expressed in this article are solely those of the authors and do not necessarily represent those of their affiliated organizations, or those of the publisher, the editors and the reviewers. Any product that may be evaluated in this article, or claim that may be made by its manufacturer, is not guaranteed or endorsed by the publisher.

## Footnotes

^1^ www.wjx.cn

## References

[ref1] Af Ursin P. Järvinen T. Pihlaja P. (2021). The role of academic buoyancy and social support in mediating associations between academic stress and school engagement in Finnish primary school children. Scand. J. Educ. Res. 65, 661–675. doi: 10.1080/00313831.2020.1739135

[ref2] Alamer A. (2021a). Basic psychological needs, motivational orientations, effort, and vocabulary knowledge: a comprehensive model. Stud. Second. Lang. Acquis. 44, 164–184. doi: 10.1017/S027226312100005X

[ref3] Alamer A. (2021b). Construct validation of self-determination theory in second language scale: a bifactor structural equation modeling approach. Front. Psychol. 12:732016. doi: 10.3389/fpsyg.2021.732016, PMID: 34659042 PMC8514625

[ref4] Alamer A. Almulhim F. (2021). The interrelation between language anxiety and self-determined motivation: a mixed methods approach. Front. Psychol. 6:619655. doi: 10.3389/feduc.2021.618655

[ref5] Al-Hoorie A. H. Oga-Baldwin W. L. Q. Hiver P. Vitta J. P. (2022). Self-determination mini-theories in second language learning: a systematic review of three decades of research. Lang. Teach. Res. 136216882211026. doi: 10.1177/13621688221102686

[ref6] Alrabai F. (2021). The influence of autonomy-supportive teaching on EFL students’ classroom autonomy: an experimental intervention. Front. Psychol. 12:728657. doi: 10.3389/fpsyg.2021.728657, PMID: 34566814 PMC8455827

[ref7] Aydın G. Michou A. (2020). Self-determined motivation and academic buoyancy as predictors of achievement in normative settings. Br. J. Educ. Psychol. 90, 964–980. doi: 10.1111/bjep.12338, PMID: 31877237

[ref8] Azadianbojnordi M. Bakhtiarpour S. Makvandi B. Ehteshamizadeh P. (2020). Can academic hope increase academic engagement in Iranian students who are university applicants? Investigating academic buoyancy as a mediator. J. Psychol. Couns. Sch., 1–9. doi: 10.1017/jgc.2020.31

[ref9] Bieleke M. Gogol K. Goetz T. Daniels L. Pekrun R. (2021). The AEQ-S: a short version of the achievement emotions questionnaire. Contemp. Educ. Psychol. 65:101940. doi: 10.1016/j.cedpsych.2020.101940

[ref10] Chaffee K. E. Noels K. A. Mceown M. S. (2014). Learning from authoritarian teachers: controlling the situation or controlling yourself can sustain motivation. Stud. Second. Lang. Learn. Teach. 4, 355–387. doi: 10.14746/ssllt.2014.4.2.9

[ref11] Chen Y. L. E. Kraklow D. (2015). Taiwanese college students’ motivation and engagement for English learning in the context of internationalization at home: a comparison of students in EMI and non-EMI programs. J. Stud. Int. Educ. 19, 46–64. doi: 10.1177/1028315314533607

[ref13] Chu W. Liu H. (2022). A mixed-methods study on senior high school EFL teacher resilience in China. Front. Psychol. 13:865599. doi: 10.3389/fpsyg.2022.865599, PMID: 35572243 PMC9094680

[ref14] Collie R. J. Ginns P. Martin A. J. Papworth B. (2017). Academic buoyancy mediates academic anxiety’s effects on learning strategies: an investigation of English and Chinese speaking Australian students. Educ. Psychol. (Lond). 37, 947–964. doi: 10.1080/01443410.2017.1291910

[ref15] Collie R. J. Martin A. J. Malmberg L. E. Hall J. Ginns P. (2015). Academic buoyancy, student’s achievement, and the linking role of control: a cross-lagged analysis of high school students. Br. J. Educ. Psychol. 85, 113–130. doi: 10.1111/bjep.12066, PMID: 25604513

[ref16] Comanaru R. Noels K. A. (2009). Self-determination, motivation, and the learning of Chinese as a heritage language. Can. Mod. Lang. Rev. 66, 131–158. doi: 10.1353/cml.0.0101

[ref17] Datu J. A. D. Yang W. (2019). Academic buoyancy, academic motivation, and academic achievement among filipino high school students. Curr. Psychol. 40, 3958–3965. doi: 10.1007/s12144-019-00358-y

[ref18] Deci E. L. Ryan R. M. (1985). Intrinsic Motivation and Self-determination in Human Behavior. New York, NY: Plenum.

[ref19] Derakhshan A. Fathi J. Pawlak M. Kruk M. (2022). Classroom social climate, growth language mindset, and student engagement: the mediating role of boredom in learning English as a foreign language. J. Multiling. Multicult. Dev., 1–19. doi: 10.1080/01434632.2022.2099407

[ref20] Derakhshan A. Kruk M. Mehdezadeh M. Pawlak M. (2021). Boredom in online classes in the Iranian EFL context: sources and solutions. System 101:102556. doi: 10.1016/j.system.2021.102556

[ref21] Dewaele J. M. Li C. (2021). Teacher enthusiasm and students’ social-behavioral learning engagement: the mediating role of student enjoyment and boredom in Chinese EFL classes. Lang. Teach. Res. 25, 922–945. doi: 10.1177/13621688211014538

[ref22] Dincer A. Yeşilyurt S. Noels K. A. Lascano D. I. V. (2019). Self-determination and classroom engagement of EFL learners: a mixed-methods study of the self-system model of motivational development. SAGE Open 9:215824401985391. doi: 10.1177/2158244019853913

[ref23] Dörnyei Z. (2009). Individual differences: interplay of learner characteristics and learning environment. Lang. Learn. 59, 230–248. doi: 10.1111/j.1467-9922.2009.00542.x

[ref24] Dörnyei Z. Ryan S. (2015). The Psychology of the Language Learner Revisited. New York: Routledge.

[ref25] Ellis R. (2008). The Study of Second Language Acquisition (2nd Edn.). Oxford: Oxford University Press.

[ref26] Fahlman S. A. Mercer-lynn K. B. Flora D. B. Eastwood J. D. (2013). Development and validation of the multidimensional state boredom scale. Assessment 20, 68–85. doi: 10.1177/1073191111421303, PMID: 21903683

[ref27] Finn J. D. Zimmer K. S. (2012). “Student engagement: what is it? Why does it matter,” in Handbook of research on student engagement. eds. Christenson S. L. Reschly A. L. Wylie C. (New York: Springer), 97–132. doi: 10.1007/978-1-4614-2018-7_5

[ref29] Gao L. Liu H. Liu X. (2022). Exploring senior high school students’ English learning demotivation in mainland China. Front. Psychol. 13:822276. doi: 10.3389/fpsyg.2022.822276, PMID: 35242085 PMC8885720

[ref30] Hayes A. F. (2018). Introduction to Mediation, Moderation, and Conditional Process Analysis: A Regression-based Approach (2nd Edn.). New York: Guilford Publications.

[ref31] Henry A. (2017). Rewarding foreign language learning: effects of the Swedish grade point average enhancement initiative on students’ motivation to learn French. Lang. Learn. 45, 301–315. doi: 10.1080/09571736.2013.853823

[ref32] Hirvonen R. Putwain D. W. Määttä S. Ahonen T. Kiuru N. (2019). The role of academic buoyancy and emotions in students' learning-related expectations and behaviours in primary school. Br. J. Educ. Psychol. 90, 948–963. doi: 10.1111/bjep.12336, PMID: 31876959

[ref33] Horwitz E. Horwitz M. Cope J. (1986). Foreign language classroom anxiety. Mod. Lang. J. 70, 125–132. doi: 10.2307/327317

[ref34] Jahedizadeh S. Ghonsooly B. Ghanizadeh A. (2019). Academic buoyancy in higher education: developing sustainability in language learning through encouraging buoyant EFL students. J. Appl. Res. High. Educ. 11, 162–177. doi: 10.1108/JARHE-04-2018-0067

[ref35] Jahedizadeh S. Ghonsooly B. Ghanizadeh A. (2021). A model of language students’ sustained flow, personal best, buoyancy, evaluation apprehension, and academic achievement. Porta Linguarum 35, 257–275. doi: 10.30827/portalin.v0i35.15755

[ref36] Joe H. Hiver P. Al-Hoorie A. H. (2017). Classroom social climate, self-determined motivation, willingness to communicate, and achievement: a study of structural relationships in instructed second language settings. Learn. Individ. Differ. 53, 133–144. doi: 10.1016/j.lindif.2016.11.005

[ref37] Khajavy G. H. MacIntyre P. D. Barabadi E. (2018). Role of the emotions and classroom environment in willingness to communicate: applying doubly latent multilevel analysis in second language acquisition research. Stud. Second. Lang. Acquis. 40, 605–624. doi: 10.1017/S0272263117000304

[ref38] Khodadady E. Khajavy G. H. (2013). Exploring the role of anxiety and motivation in foreign language achievement: a structural equation modeling approach. Porta Linguarum 20, 269–286. doi: 10.30827/Digibug.20240

[ref39] Kong L. C. Liu W. C. (2020). Understanding motivational profiles of high-ability female students from a Singapore secondary school: a self-determination approach. Asia-Pac. Educ. Res. 29, 529–539. doi: 10.1007/s40299-020-00504-2

[ref40] Lei L. Liu D. (2021). Conducting Sentiment Analysis (Elements in Corpus Linguistics). Cambridge: Cambridge University Press.

[ref41] Li H. Liu H. (2021). Beginning EFL teachers’ emotional labor strategies in the Chinese context. Front. Psychol. 12:737746. doi: 10.3389/fpsyg.2021.737746, PMID: 34489840 PMC8418064

[ref42] Lin Y. (2019). Taiwanese EFL learners' willingness to communicate in English in the classroom: impacts of personality, affect, motivation, and communication confidence. Asia-Pac. Educ. Res. 28, 101–113. doi: 10.1007/s40299-018-0417-y

[ref43] Liu H. Chu W. (2022). Exploring EFL teacher resilience in the Chinese context. System 105:102752. doi: 10.1016/j.system.2022.102752

[ref44] Liu H. Han X. (2022). Exploring senior high school students’ English academic resilience in the Chinese context. Chin. J. Appl. Linguist. 45, 49–68. doi: 10.1515/cjal-2022-0105

[ref45] Liu H. Song X. (2021). Exploring “flow,” in young Chinese EFL learners’ online English learning activities. System 96:102425. doi: 10.1016/j.system.2020.102425

[ref46] Liu W. C. Wang J. C. K. Kang H. J. Kee Y. H. (2021). A motivation profile analysis of Malay students in Singapore. Asia Pac. J. Educ. 41, 299–311. doi: 10.1080/02188791.2020.1770690

[ref47] Liu H. Yan C. Fu J. (2022). Exploring livestream English teaching anxiety in the Chinese context: an ecological perspective. Teach. Teach. Educ. 111:103620. doi: 10.1016/j.tate.2021.103620

[ref48] Lou N. M. Noels K. A. (2021). Western and heritage cultural internalizations predict EFL students’ language motivation and confidence. Int. J. Biling. Educ. Biling. 24, 636–650. doi: 10.1080/13670050.2018.1508277

[ref49] MacIntyre P. D. Gardner R. C. (1994). The subtle effects of language anxiety on cognitive processing in the second language. Lang. Learn. 44, 283–305. doi: 10.1111/j.1467-1770.1994.tb01103.x

[ref50] MacIntyre P. Mercer S. (2014). Introducing positive psychology to SLA. Stud. Second Lang. Learn. Teach. 4, 153–172. doi: 10.14746/ssllt.2014.4.2.2

[ref51] Malmberg L. E. Hall J. Martin A. J. (2013). Academic buoyancy in secondary school: exploring patterns of convergence in English, mathematics, science, and physical education. Learn. Individ. Differ. 23, 262–266. doi: 10.1016/j.lindif.2012.07.014

[ref52] Martin A. J. (2014). Academic buoyancy and academic outcomes: towards a further understanding of students with attention-deficit/hyperactivity disorder (ADHD), students without ADHD, and academic buoyancy itself. Br. J. Educ. Psychol. 84, 86–107. doi: 10.1111/bjep.12007, PMID: 24547755

[ref53] Martin A. J. Colmar S. H. Davey L. A. Marsh H. W. (2010). Longitudinal modelling of academic buoyancy and motivation: do the '5Cs' hold up over time. Br. J. Educ. Psychol. 80, 473–496. doi: 10.1348/000709910X486376, PMID: 20170601

[ref54] Martin A. J. Marsh H. W. (2008). Academic buoyancy: towards an understanding of students’ every day academic resilience. J. Sch. Psychol. 46, 53–83. doi: 10.1016/j.jsp.2007.01.002, PMID: 19083351

[ref55] Noels K. (2001). Learning Spanish as a second language: Learners' orientations and perceptions of their teachers' communication style. Lang. Learn. 51, 107–144. doi: 10.1111/0023-8333.00149

[ref56] Noels K. Clément R. Pelletier L. (1999). Perceptions of teachers’ communicative style and students’ intrinsic and extrinsic motivation. Mod. Lang. J. 83, 23–34. doi: 10.1111/0026-7902.00003

[ref57] Noels K. A. Lou N. M. Vargas Lascano D. I. Chaffee K. E. Dincer A. Zhang Y. S. D. (2020). “Self-determination and motivated engagement in language learning” in The Palgrave Handbook of Motivation for Language Learning. eds. Lamb M. Csizér K. Henry A. Ryan S. (Cham: Palgrave Macmillan).

[ref58] Noels K. A. Pelletier L. G. Clément R. Vallerand R. J. (2000). Why are you learning a second language? Motivational orientations and self-determination theory. Lang. Learn. 50, 57–85. doi: 10.1111/0023-8333.00111

[ref60] Oga-Baldwin W. L. Q. Nakata Y. (2017). Engagement, gender, and motivation: a predictive model for Japanese young language learners. System 65, 151–163. doi: 10.1016/j.system.2017.01.011

[ref61] Oxford R. (2016). “Toward a psychology of well-being for language learners: the ‘empathics’ vision,” in The Positive Psychology in SLA. eds. MacIntyre P. D. Gregersen T. Mercer S. (Bristol: Multilingual Matters).

[ref62] Pae T. I. Shin S. K. (2011). Examining the effects of differential instructional methods on the model of foreign language achievement. Learn. Individ. Differ. 21, 215–222. doi: 10.1016/j.lindif.2010.11.023

[ref63] Pavlenko A. (2013). “The affective turn in SLA: from ‘affective factors’ to ‘language desire’ and ‘commodification of affect’,” in The Affective Dimension in Second Language Acquisition. eds. Gabryś D. Bielska J. (Bristol: Multilingual Matters).

[ref64] Pawlak M. Kruk M. Zawodniak J. Pasikowski S. (2020c). Investigating factors responsible for boredom in English classes: the case of advanced learners. System 91:102259. doi: 10.1016/j.system.2020.102259

[ref65] Pawlak M. Zawodniak J. Kruk M. (2020a). The neglected emotion of boredom in teaching English to advanced learners. Int. J. Appl. Linguist. 30, 497–509. doi: 10.1111/ijal.12302

[ref66] Pawlak M. Zawodniak J. Kruk M. (2020b). Individual trajectories of boredom in learning English as a foreign language at the university level: insights from three students’ self-reported experience. Innov. Lang. Learn. Teach. 15, 263–278. doi: 10.1080/17501229.2020.1767108

[ref67] Pekrun R. Goetz T. Frenzel A. C. Barchfeld B. Perry R. P. (2011). Measuring emotions in students’ learning and performance: the achievement emotions questionnaire (AEQ). Contemp. Educ. Psychol. 36, 36–48. doi: 10.1016/j.cedpsych.2010.10.002

[ref68] Pekrun R. Goetz T. Perry R. P. (2005). Academic Emotions Questionnaire (AEQ): User's Manual. Munich, Germany: University of Munich, Department of Psychology.

[ref69] Pelletier L. G. Fortier M. S. Vallerand R. J. Brière N. M. (2001). Associations among perceived autonomy support, forms of self-regulation, and persistence: a prospective study. Motiv. Emot. 25, 279–306. doi: 10.1023/A:1014805132406

[ref70] Peng J. Woodrow L. (2010). Willingness to communicate in English: a model in Chinese EFL classroom context. Lang. Learn. 60, 834–876. doi: 10.1111/j.1467-9922.2010.00576.x

[ref71] Putwain D. W. Daly A. L. Chamberlain S. Sadreddini S. (2015). Academically buoyant students are less anxious about and perform better in high-stakes examinations. Br. J. Educ. Psychol. 85, 247–263. doi: 10.1111/bjep.12068, PMID: 25739681

[ref72] Putwain D. W. Gallard D. Beaumont J. (2020). Academic buoyancy protects achievement against minor academic adversities. Learn. Individ. Differ. 83-84, 101936–101984. doi: 10.1016/j.lindif.2020.101936

[ref73] Putwain D. W. Wood P. Pekrun R. (2022). Achievement emotions and academic achievement: reciprocal relations and the moderating influence of academic buoyancy. J. Educ. Psychol. 114, 108–126. doi: 10.1037/edu0000637

[ref74] Reeve J. Tseng C. (2011). Agency as a fourth aspect of students’ engagement during learning activities. Contemp. Educ. Psychol. 36, 257–267. doi: 10.1016/j.cedpsych.2011.05.002

[ref75] Ross A. (2015). An exploration of the emotions and motivation of tertiary English language learners in Australia. Ph.D. thesis. Canberra: University of Canberra.

[ref76] Ryan R. M. Connell J. P. (1989). Perceived locus of causality and internalization: examining reasons for acting in two domains. J. Pers. Soc. Psychol. 57, 749–761. doi: 10.1037//0022-3514.57.5.749, PMID: 2810024

[ref77] Ryan R. M. Deci E. L. (2000). Self-determination theory and the facilitation of intrinsic motivation, social development, and well-being. Am. Psychol. 55, 68–78. doi: 10.1037//0003-066x.55.1.68, PMID: 11392867

[ref78] Ryan R. M. Deci E. L. (2020). Intrinsic and extrinsic motivation from a self-determination theory perspective: definitions, theory, practices, and future directions. Contemp. Educ. Psychol. 61:1018600:101860. doi: 10.1016/j.cedpsych.2020.101860

[ref80] Shao K. Parkinson B. (2021). Social psychological accounts of peer emotion transfer in EFL classrooms: a doubly latent multilevel analysis. Lang. Teach. Res.:136216882110115. doi: 10.1177/13621688211011513

[ref81] Shao K. Pekrun R. Marsh H. W. Loderer K. (2020). Control-value appraisals, achievement emotions, and foreign language performance: a latent interaction analysis. Learn. Instr. 69:101356. doi: 10.1016/j.learninstruc.2020.101356

[ref82] Shao K. Pekrun R. Nicholson L. J. (2019). Emotions in classroom language learning: what can we learn from achievement emotion research? System 86:102121. doi: 10.1016/j.system.2019.102121

[ref83] Shao K. Yu W. Ji Z. (2013). An exploration of Chinese EFL students’ emotional intelligence and foreign language anxiety. Mod. Lang. J. 97, 917–929. doi: 10.1111/j.1540-4781.2013.12042.x

[ref84] Shelton-Strong S. J. (2020). Advising in language learning and the support of learners’ basic psychological needs: a self-determination theory perspective. Lang. Teach. Res. 26, 963–985. doi: 10.1177/1362168820912355

[ref85] Shirvan M. E. Alamer A. (2022). Modeling the interplay of EFL learners' basic psychological needs, grit and L2 achievement. J. Multiling. Multicult. Dev., 1–17. doi: 10.1080/01434632.2022.2075002

[ref86] Shirvan M. E. Yazdanmehr E. Taherian T. Kruk M. Pawlak M. (2021). Boredom in practical English language classes: a longitudinal confirmatory factor analysis-curve of factors model. Appl. Linguist. Rev. doi: 10.1515/applirev-2021-0073

[ref87] Sugita-McEown M. Oga-Baldwin W. L. Q. (2019). Self-determination for all language learners: new applications for formal language education. System 86:102124. doi: 10.1016/j.system.2019.102124

[ref88] Swain M. (2013). The inseparability of cognition and emotion in second language learning. Lang. Teach. 46, 195–207. doi: 10.1017/S0261444811000486

[ref89] Takahashi C. Seongah I. (2020). Comparing self-determination theory and the L2 motivational self system and their relationships to L2 proficiency. Stud. Second Lang. Learn. Teach. 10, 673–696. doi: 10.14746/ssllt.2020.10.4.2

[ref90] Thomas C. L. Allen K. (2021). Driving engagement: investigating the influence of emotional intelligence and academic buoyancy on student engagement. J. Furth. High. Educ. 45, 107–119. doi: 10.1080/0309877X.2020.1741520

[ref91] Tsao J. Tseng W. Hsiao C. Wang C. Gao X. (2021). Toward a motivation-regulated learner engagement WCF model of L2 writing performance. SAGE Open 11, 215824402110231–215824402110213. doi: 10.1177/21582440211023172

[ref001] Vallerand R. J. Blais M. R. Brière N. M. Pelletier L. G. (1989). Construction and validation of the Academic Motivation Scale. Can. J. Behav. Sci. 21, 323–349.

[ref92] Wang X. Liu Y. L. Ying B. Lin J. (2021). The effect of learning adaptability on Chinese middle school students’ English academic engagement: the chain mediating roles of foreign language anxiety and English learning self-efficacy. Curr. Psychol. doi: 10.1007/s12144-021-02008-8

[ref93] Yang S. Noughabi M. A. Jahedizadeh S. (2022). Modelling the contribution of English language learners' academic buoyancy and self-efficacy to L2 grit: evidence from Iran and China. J. Multiling. Multicult. Dev., 1–17. doi: 10.1080/01434632.2022.2062368

[ref94] Yun S. Hiver P. Al-Hoorie A. H. (2018). Academic buoyancy: exploring learners’ everyday resilience in the language classroom. Stud. Second. Lang. Acquis. 40, 805–830. doi: 10.1017/S0272263118000037

[ref95] Zhang X. Dai S. Ardasheva Y. (2020). Contributions of (de)motivation, engagement, and anxiety to English listening and speaking. Learn. Individ. Differ. 79:101856. doi: 10.1016/j.lindif.2020.101856

[ref96] Ryan R. M. Deci E. L. (2017). Self-Determination Theory: Basic Psychological Needs in Motivation, Development, and Wellness. New York: The Guilford PressProvide the volume number for “Yang et al., 2022.”

